# A screen for mutants with abnormal cilia number in *C. elegans*

**DOI:** 10.1186/2046-2530-1-S1-P61

**Published:** 2012-11-16

**Authors:** T Adachi

**Affiliations:** 1 Kanagawa University, Japan

## 

To date, while molecular basis of intraflagellar transport (IFT) has been well studied, little is known about the mechanisms underlying the early phase of ciliogenesis. In *Caenorhabditis elegans*, sensory neurons are associated with one or two cilia. The number is not stochastically but strictly determined according to the cell fate. For example, one of the head sensory neuron class, ADL, is double ciliated, whereas another sensory neuron class, ASE, is single ciliated. To dissect molecular mechanisms that determine the number of cilia, we screen for mutants with abnormal cilia number. Through the screening, we obtained three mutants, in which ADL is single ciliated. In these mutants, ADL cilium did not display short-length phenotype, which is typically observed in IFT-defective mutants. Currently, we are mapping the mutations responsible for the abnormal cilia number phenotype.

